# Two staging systems for gastrointestinal stromal tumors in the stomach: which is better?

**DOI:** 10.1186/s12876-017-0705-7

**Published:** 2017-12-06

**Authors:** Chul Hong Park, Gwang Ha Kim, Bong Eun Lee, Geun Am Song, Do Youn Park, Kyung Un Choi, Dae Hwan Kim, Tae Yong Jeon

**Affiliations:** 1 Department of Internal Medicine, Pusan National University School of Medicine, and Biomedical Research Institute Pusan National University Hospital, 179, Gudeok-ro, Seo-Gu, Busan, 49241 South Korea; 2 Department of Pathology, Pusan National University School of Medicine, Busan, South Korea; 3 Department of Surgery, Pusan National University School of Medicine, Busan, South Korea

**Keywords:** Stomach, Gastrointestinal stromal tumors, Staging, Prognosis, Recurrence

## Abstract

**Background:**

The prognosis of a gastrointestinal stromal tumor (GIST) is influenced by its anatomic site; however, few studies on the prognosis of gastric GISTs have been reported. The aims of this study were to evaluate long-term prognoses of patients who underwent surgical resection for gastric GISTs and to compare the clinical efficacy of two staging systems: the National Institutes of Health (NIH) consensus criteria and the 7th Union for International Cancer Control/American Joint Committee on Cancer (UICC/AJCC) tumor-node-metastasis (TNM) staging system.

**Methods:**

We conducted a retrospective observational study of 145 patients who underwent surgical resection for gastric GISTs between February 2001 and June 2012 at Pusan National University Hospital (Busan, Korea). Recurrence and 5-year recurrence-free survival (RFS) rates were analyzed.

**Results:**

During a median follow-up period of 44 months (range, 6–144 months), 11 recurrent lesions were detected in 9 patients (6.4%). On multivariate analysis, tumor size (>5 cm), mitotic count (>5/50 high-power fields), and epithelioid and mixed pathological type were significantly associated with recurrence. The overall 5-year RFS rate was 93.4%. Although no statistically significant differences were detected (C-statistic difference *P* = 0.886), all metrics showed lower values for the UICC/AJCC TNM staging system than for the NIH consensus criteria, suggesting that the UICC/AJCC TNM staging system may be a better model.

**Conclusions:**

The 5-year RFS rate in patients who underwent curative resection for gastric GISTs was excellent. The UICC/AJCC TNM staging system may be more useful than the NIH consensus criteria for risk categorization of patients with gastric GISTs.

## Background

Gastrointestinal stromal tumors (GISTs) are the most common mesenchymal tumors of the gastrointestinal tract. GISTs originate from intestinal pacemaker cells, known as interstitial cells of Cajal [1]. The pathogenesis of GISTs is related to oncogenic mutations in either the *KIT* or *PDGFR* ***α*** genes [2, 3]. GISTs can occur anywhere along the gastrointestinal tract. The most common site is the stomach (60%), followed by the jejunum and ileum (30%), duodenum (4–5%), rectum (4%), colon and appendix (1–2%), and esophagus (<1%) [4]. These tumors rarely occur outside the gastrointestinal tract at sites such as the omentum, mesentery, or retroperitoneum [4].

GISTs are characterized by a diverse spectrum of morphological and clinical features ranging from benign to malignant. Approximately 10–30% of GISTs are clinically malignant, but all GISTs are considered to have some degree of malignant potential [5]. The primary treatment for localized GISTs is complete surgical resection with microscopic negative margins. Patients with unresectable, metastatic, or recurrent GISTs can be treated with imatinib mesylate. Moreover, adjuvant treatment with imatinib mesylate after surgical resection is proven to improve recurrence-free survival (RFS) of patients with GISTs [6–8].

To assess risk in the clinical course of GISTs, a consensus conference held at the National Institutes of Health (NIH) in 2001 provided the first practical scheme for risk categorization based on tumor size and mitotic index (per 50 high-power fields [HPF]) [2]. Since that time, the NIH consensus criteria have been most commonly used in clinical studies. However, these criteria do not consider the primary location of GISTs in predicting risk. Therefore, based on the different clinical outcomes of GISTs according to primary tumor site, the 7th Union for International Cancer Control/American Joint Committee on Cancer (UICC/AJCC) tumor-node-metastasis (TNM) staging system was proposed in 2010 [9].

Generally, the prognoses of GISTs are influenced by their anatomic sites. It has been reported that patients with gastric GISTs have better prognoses than those with non-gastric GISTs [4, 10–12]. While many studies have reported prognoses of all GISTs irrespective of their primary anatomic sites, studies specific to gastric GISTs are few in number. A recent retrospective multicenter study of 1057 gastric GISTs suggested that Eastern patients with gastric GISTs had more favorable outcomes than Western patients [13]. Therefore, we aimed to investigate the long-term prognoses of patients with gastric GISTs who underwent curative surgical resection and to compare the clinical efficacy of the 7th UICC/AJCC TNM staging system with that of the NIH consensus criteria.

## Methods

### Patients

The study cohort consisted of 155 patients who underwent surgery for gastric GISTs at Pusan National University Hospital (Busan, Korea) between February 2001 and June 2012. The inclusion criterion was patients who underwent curative surgical resection for primary gastric GISTs. The exclusion criteria were distant metastasis at the time of surgery, preoperative chemotherapy with imatinib and the presence of another concomitant malignancy. Ten patients were excluded because they were incidentally discovered to have gastric GISTs during gastrectomy for gastric cancer. Finally, 145 patients with gastric GISTs were included in this study. Written informed consent was obtained from all patients, and the study protocol was reviewed and approved by the Institutional Review Board of Pusan National University Hospital (E − 2015216).

### Histopathology

**Fig. 1:**
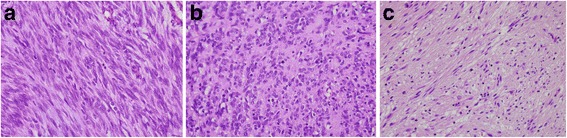
Histologic subtypes of GISTs. **a** Spindle cell type. The tumor is composed of fusiform spindle cells with a fascicular growth pattern. **b** Epithelioid type. The tumor is composed of large round or polygonal cells with abundant, often eosinophilic or clear cytoplasm. **c** Mixed type. The tumor is composed of mixture of both types (hematoxylin and eosin staining, original magnification x400).

All resected specimens were examined by two expert pathologists (DY Park and KU Choi). Paraffin-embedded resected specimens were sectioned and stained with hematoxylin and eosin. A diagnosis of GIST was confirmed by immunohistochemical staining for c-Kit (CD117) or DOG-1 and CD34. Histologic features such as tumor location, tumor size, mitotic count, or histologic subtype (spindle, epithelioid, or mixed) were also evaluated (Fig. 1). A tumor’s size was defined as its largest diameter. The mitotic count of a tumor was evaluated as the number of mitosis per 50 HPF. In discordant cases, the histopathologic findings were adjudicated by consensus between the two pathologists.

Tumor staging was performed according to the NIH consensus criteria and the 7th UICC/AJCC TNM staging system. Under the NIH consensus criteria, tumors were categorized as very low risk, low risk, intermediate risk, and high risk based on mitotic activity and tumor size, as shown in Table 1 [2]. Under the 7th UICC/AJCC TNM staging system, tumors were categorized into 4 T-categories based solely on tumor size; the T-category was then associated with the mitotic rate. The presence of either nodal or distant metastasis indicates stage IV disease [9].

**Table 1:** The NIH consensus criteria and the 7th UICC/AJCC TNM staging system for gastric GISTs

Tumor size (cm)	Mitotic count (/50 HPF)	NIH criteria	UICC/AJCC TNM stage^a^
≤2	≤5	Very low	IA
>2, ≤5	≤5	Low	IA
>5, ≤10	≤5	Intermediate	IB
>10	≤5	Intermediate	II
≤2	>5	Intermediate	II
>2, ≤5	>5	Intermediate	II
>5, ≤10	>5	High	IIIA
>10	>5	High	IIIB

*HPF* high-power fields

^a^All tumors with lymph node or other metastasis are classified as stage IV

### Follow-up

Postoperative follow-up information was obtained at the regular outpatient visits. Each patient’s medical history was obtained, and all underwent a physical examination, esophagogastroduodenoscopy, and abdominal pelvic computed tomography 6 months after surgery and annually thereafter. All patients underwent the same follow-up protocol regardless of risk stratification. A diagnosis of recurrence was based on clinical, radiologic or endoscopic findings.

### Statistical analyses

Categorical variables are expressed as a percentage and continuous variables are expressed by median and range. Tumor size was divided into two groups (≤5 cm and >5 cm), and mitotic count was grouped into two categories (≤5/50 HPF and >5/50 HPF). Univariate and multivariate survival analyses included demographic and clinical variables such as sex, age, tumor size, mitotic count, histologic subtype, or operative method. Hazard ratios (HRs) and 95% confidence intervals (CIs) were calculated using the Cox proportional hazard model. RFS was defined as the interval between the date of surgery and either the date of the last follow-up or the date of recurrence, and was calculated by using the Kaplan-Meier method. Model fit statistics such as the −2 log likelihood ratio, Akaike’s Information Criterion (AIC), or Schwarz Bayesian Criterion (SBC) were used to compare the two different risk stratification methods. The better model was indicated by a lower value of each statistic. A *P* value <0.05 was regarded statistically significant. All data were analyzed by the SAS software, version 9.3 (SAS Institute, Cary, NC, USA) and R software, version 3.2.2 (R Foundation for Statistical Computing, Vienna, Austria).

## Results

### Clinicopathologic characteristics of patients with gastric GISTs

Clinicopathologic characteristics of the 145 patients who underwent surgical resection for gastric GISTs are summarized in Table 2. The patients included 64 men and 81 women with a median age of 59 years (range, 4–79 years). Eighty-one tumors were located in the upper third of the stomach, 52 in the middle third, and 12 in the lower third. GISTs were resected via laparoscopic surgery in 86 patients and open surgery in 59 patients. Complete resection was achieved in 144 patients (99.3%); only one case had a ruptured GIST at the time of diagnosis. The median tumor size was 3.1 cm (range, 0.7–26 cm). Most tumors (120/145; 82.8%) were ≤5 cm in maximum diameter. The mitotic figure count was ≤5/50 HPF in 98 tumors, >5/50 HPF and ≤10/50 HPF in 25 tumors, and >10/50 HPF in 22 tumors. The predominant histologic subtype was spindle cell type (130/145, 89.7%). Six patients (4.1%) were administered imatinib mesylate as adjuvant treatment.

**Table 2:** Baseline clinicopathologic characteristics of 145 patients who underwent surgical resection for gastric GISTs

	No. of patients (%)
Median age, years (range)	59 (4–79)
Sex, *n* (%)	
Male	64 (44.1)
Female	81 (55.9)
Tumor location, *n* (%)	
Upper third	81 (55.9)
Middle third	52 (35.9)
Lower third	12 (8.3)
Median tumor size, cm (range)	3.1 (0.7–26)
Tumor size (cm), *n* (%)	
≤ 2	29 (20.0)
> 2, ≤5	91 (62.8)
> 5, ≤10	17 (11.7)
> 10	8 (5.5)
Mitotic count (per 50 high-power fields), *n* (%)	
≤ 5	98 (67.6)
> 5, ≤10	25 (17.2)
> 10	22 (15.2)
Histologic subtype, *n* (%)	
Spindle type	130 (89.7)
Epithelioid type	13 (9.0)
Mixed type	2 (1.4)
Operative method, *n* (%)	
Laparoscopic surgery	86 (59.3)
Open surgery	59 (40.7)

According to the NIH consensus criteria, 25 patients (17.2%) were in the very low risk group, 64 (44.1%) in the low risk group, 23 (15.9%) in the intermediate risk group, and 33 (22.8%) in the high risk group. According to the 7th UICC/AJCC TNM staging system, 89 patients (61.4%) were stage IA, 7 (4.8%) were IB, 34 (23.4%) were II, 10 (6.9%) were IIIA, and 5 (3.4%) were IIIB.

### Recurrence

During the median follow-up period of 44 months (range, 6–144 months), 9 patients (6.2%) experienced recurrence; of these patients, recurrence was histopathologically confirmed in 3. The most common site was the liver (45.4%), followed by the peritoneal cavity (27.3%), site of surgery (18.2%), and spleen (9.1%) (Table 3). According to the NIH consensus criteria, all nine cases were in the intermediate or high risk groups; recurrence was observed in 3 (13.0%) of 23 intermediate risk cases and 6 (18.2%) of 33 high risk cases. According to the 7th UICC/AJCC TNM staging system, tumor recurrence occurred in one stage I case, three stage II cases, four stage IIIA cases, and one stage IIIB case. The recurrence rates at each stage were 1.0% in stage I, 8.8% in stage II, 40.0% in stage IIIA, and 20.0% in stage IIIB. All nine patients were treated with imatinib mesylate after recurrent disease was confirmed.

**Table 3:** Long-term outcomes of 145 patients who underwent surgical resection for gastric GISTs

	No. of patients (%)
Median follow-up duration, month (range)	44 (6–144)
Recurrence, *n* (%)	
Yes	9 (6.2)
No	136 (93.8)
Recurrence site^a^, *n* (%)	
Liver	5 (45.5)
Peritoneum	3 (27.3)
Spleen	2 (18.2)
Operation site	1 (9.1)
Recurrence according to NIH consensus criteria	
Very low	0/25 (0)
Low	0/65 (0)
Intermediate	3/23 (13.0)
High	6/33 (18.2)
Recurrence according to 7th UICC/AJCC TNM staging system	
I	1/96 (1.0)
II	3/34 (8.8)
IIIA	4/10 (40)
IIIB	1/5 (20)

^a^11 recurrent lesions were observed in 9 patients

### Factors associated with recurrence in patients with gastric GISTs

Univariate analysis revealed that tumor size (>5 cm), mitotic count (>5/50 HPF), and histologic subtype (epithelioid and mixed type) were associated with recurrence (Table 4). When all six variables were included in the Cox proportional hazard regression model, tumor size >5 cm (HR, 10.75; 95% CI, 2.12–54.60; *P* = 0.004), mitotic count >5/50 HPF (HR, 10.55; 95% CI, 1.25–88.85; *P* = 0.030), and epithelioid and mixed type (HR, 5.73; 95% CI, 1.29–25.53; *P* = 0.022) were significantly associated with recurrence via multivariate analyses (Table 4).

**Table 4:** Factors associated with recurrence in patients who underwent surgical resection for gastric GISTs

Variables	Univariate Analysis			Multivariate Analysis		
	HR	95% CI	*P*-value	HR	95% CI	*P*-value
Sex						
Male	1					
Female	0.58	0.16–2.17	0.420			
Age (years)						
≤ 60	1					
> 60	2.64	0.66–10.54	0.171			
Tumor size (cm)						
≤ 5	1			1		
> 5	16.58	3.44–79-83	<0.001	10.75	2.12–54.60	0.004
Mitotic count (per 50 high-power fields)						
≤ 5	1			1		
> 5	16.72	2.09–133.95	0.008	10.55	1.25–88.85	0.030
Histologic subtype						
Spindle type	1			1		
Epithelioid and mixed type	4.48	1.12–18.00	0.034	5.73	1.29–25.53	0.022
Operative method						
Laparoscopic surgery	1					
Open surgery	4.76	0.99–22.93	0.052			

*HR* hazard ratio, *CI* confident interval

### 5-year recurrence-free survival rates in patients with gastric GISTs

**Fig. 2:**
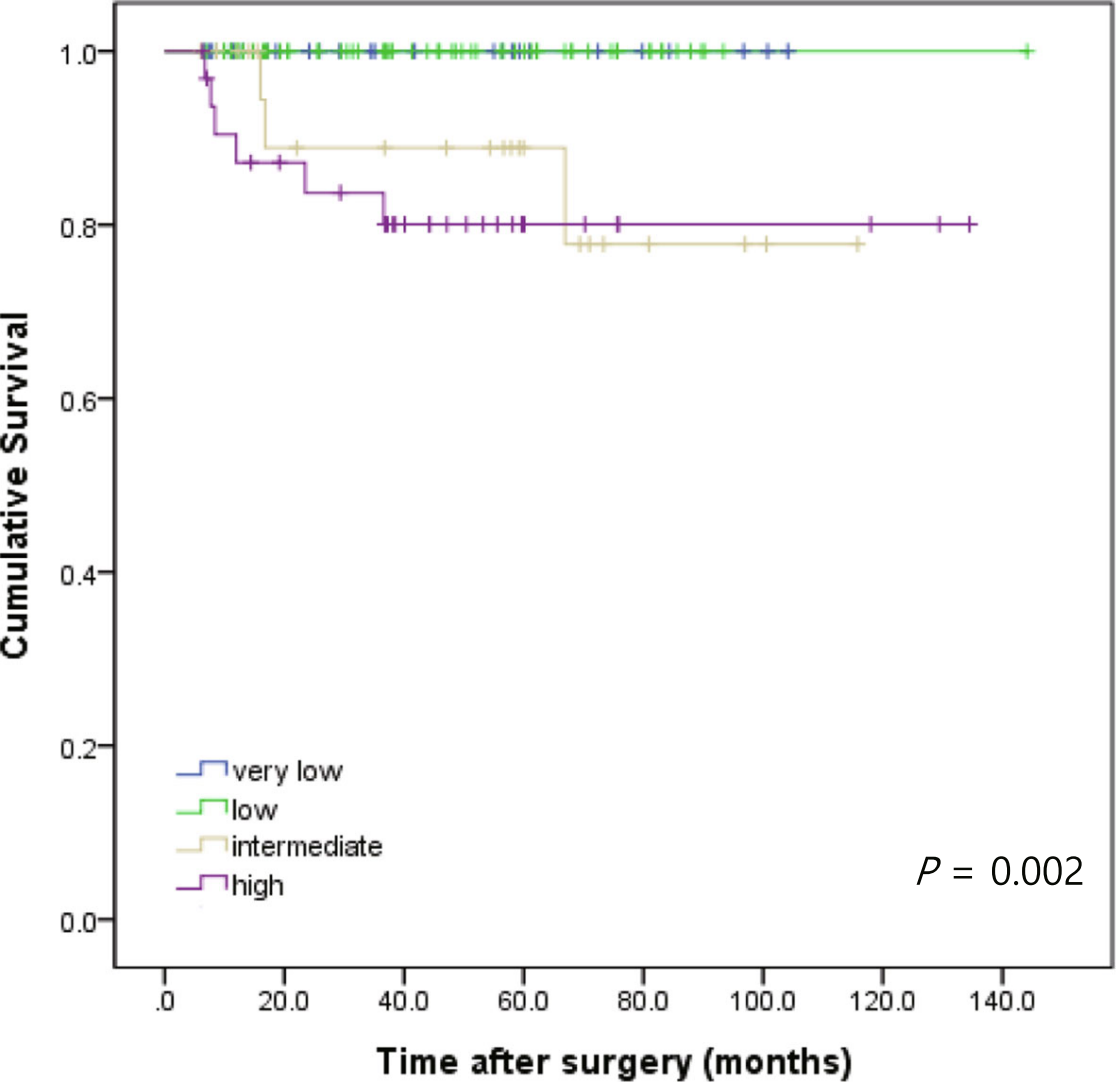
Recurrence-free survival rate of patients undergoing surgical resection for gastric GISTs according to the NIH consensus criteria

**Fig. 3:**
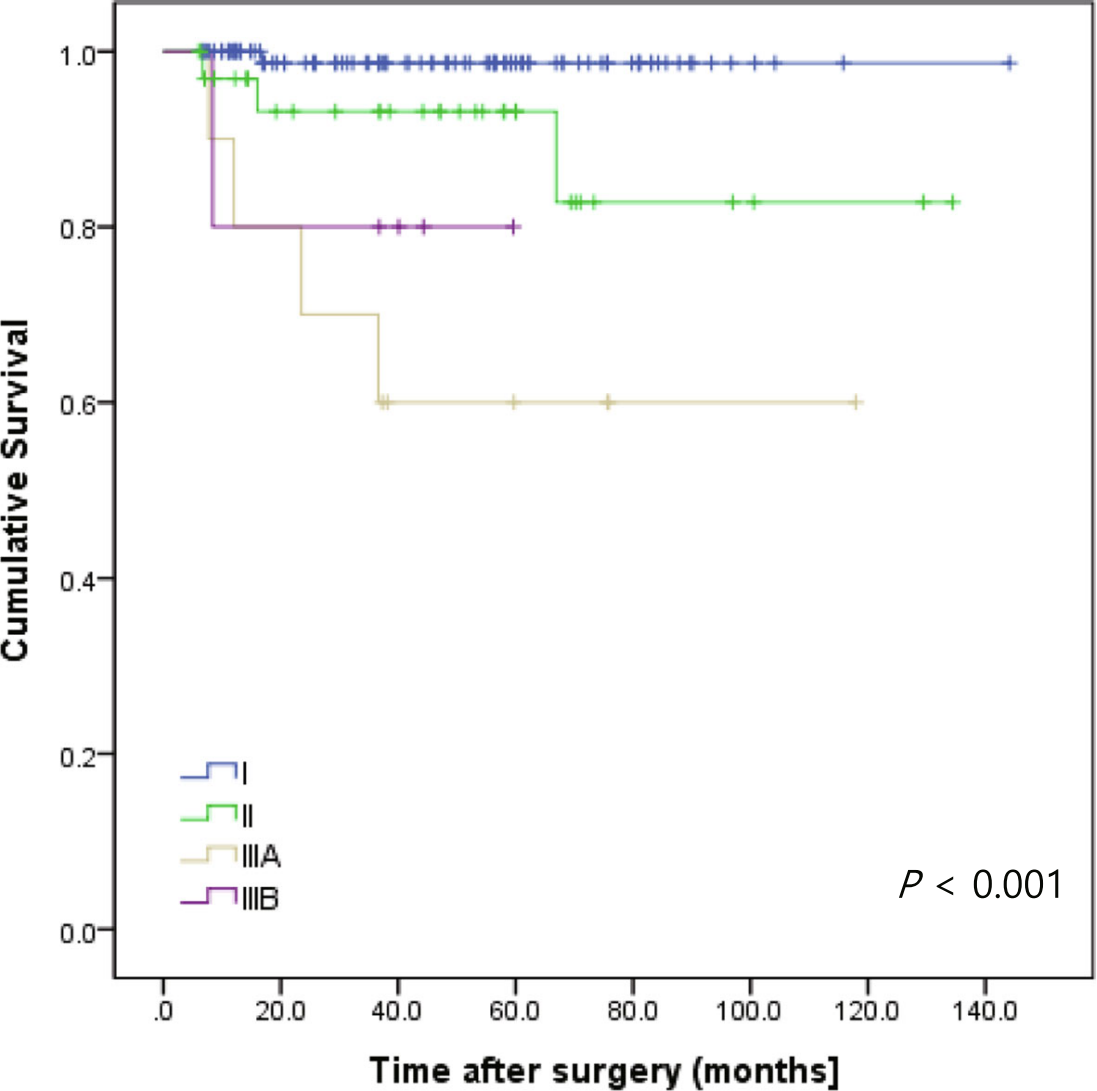
Recurrence-free survival rate of patients undergoing surgical resection for gastric GISTs according to the 7^th^ UICC/AJCC TNM staging system

The overall 5-year RFS rate was 93.4%. According to the NIH consensus criteria, 5-year RFS rates were 100.0% in the very low and low risk groups, 88.9% in the intermediate risk group, and 80.1% in the high risk group (Fig. 2). According to the 7th UICC/AJCC TNM staging system, 5-year RFS rates were 98.7% in stage I, 93.1% in stage II, 74.1% in stage IIIA and 60% in stage IIIB (Fig. 3).

### Comparison of 5-year recurrence-free survival rates according to the NIH consensus criteria and the 7th UICC/AJCC TNM staging system

The 5-year RFS rates were at least 93% in the stage I and II group by the 7th UICC/AJCC TNM staging system and were above 89% in the very low, low, and intermediate risk groups by the NIH consensus criteria. Stage III or high risk groups showed lower RFS rates. Two risk stratification classifications were redistributed to the high risk and low risk groups. The high risk group included high risk according to the NIH consensus criteria and stage IIIA and IIIB according to the 7th UICC/AJCC TNM staging system; the low risk group included very low, low, and intermediate risk according to the NIH classification and stage I and II according to the 7th UICC/AJCC TNM staging system. Accordingly, model fit statistics including the −2 log likelihood ratio, AIC and SBC were used to compare the two risk stratification models. Although no statistically significant difference was detected (C-statistic difference *P* = 0.886), all three metrics showed lower values for the 7th UICC/AJCC TNM staging system than for the NIH consensus criteria, indicating that the 7th UICC/AJCC TNM staging system may be a better model (Table 5).

**Table 5:** Comparison of the NIH consensus criteria and the 7th UICC/AJCC TNM staging system in predicting recurrence free survival in patients who underwent surgical resection for gastric GISTs

	Unadjusted	Model Fit Statistics			
	HR (95% CI)	-2 Log Likelihood	AIC	SBC	C-statistic (95% CI)^a^
NIH (high/very low-intermediate)	7.041 (1.755–28.248)	75.489	77.489	77.687	0.758 (0.615–0.901)
AJCC (III/I-II)	10.579 (2.835–39.482)	72.618	74.618	74.816	0.745 (0.639–0.851)

*AIC* Akaike’s Information Criterion, *SBC* Schwarz Bayesian Criterion

^a^Overall adequacy of risk prediction procedures with censored survival data

## Discussion

In the present study, the 5-year RFS rate in patients who underwent surgery for gastric GISTs was excellent. This was influenced significantly by tumor size, mitotic count, and histologic subtype. No differences were detected in distributions of risk groups predicting the 5-year RFS rates between the NIH consensus criteria and 7th UICC/AJCC TNM staging system. To our knowledge, this is the first study to compare these two staging systems in patients with gastric GISTs.

Although many previous studies have reported the clinical outcomes of GISTs, few studies have focused on gastric GISTs alone. Miettinen et al. first published a large-scale, retrospective study of 1765 patients with gastric GISTs, reporting on their clinicopathologic features and improved prognoses [14]. Thereafter, several studies have shown that the tumor site should be included for risk stratification of GISTs in addition to the tumor size and mitotic count of the primary tumor [4, 9]. In particular, the prognosis for gastric GISTs is significantly better than for non-gastric GISTs [4, 9, 12]. The 5-year disease free survival rate of patients with gastric GISTs is 67.5%, which is higher than that of patients with non-gastric GISTs (36.5%) [12].

In the present study, the recurrence rate of gastric GISTs after surgery was 6.2%, which is lower than the recurrence rates of 17 to 24% reported in previous Western studies [15, 16]. On the other hand, the recurrence rate in our study is similar to the recurrence rate of 2.7 to 8.9% in recent studies including Korean and Japanese patients with gastric GISTs [13, 17]. On the basis of these results, Eastern patients with gastric GISTs may have more favorable outcomes than Western patients. The common sites of recurrence in the present study were the liver and peritoneal cavity, similar to those observed in previous studies [13, 15–17].

In the present study, multivariate analyses revealed that tumor size, mitotic count, and histologic subtype were associated with recurrence after curative resection for gastric GISTs. Most studies have shown that the most important prognostic factors for GISTs are tumor size and mitotic count [14, 18, 19]. In the case of histologic subtype, some studies have reported that recurrence is not associated with histologic subtype [20, 21]. On the other hand, another study showed that patients with spindle cell type GIST show significantly higher 5-year RFS than those with epithelioid or mixed type, which is similar to our results [22].

The median age at the time of diagnosis was 59 years, and the median tumor size was 3.1 cm in our study. Compared with Western studies [14, 23, 24], patients with gastric GISTs in our study were younger and had smaller tumors. Eastern studies conducted in Korea and Japan showed similar results to our study [13, 17]. These observed differences between Western and Eastern patients might be attributable to nationwide gastric cancer screening programs for people aged 40 years and older every 2 years provided by the Korea and Japan governments [25, 26]. Therefore, Eastern patients with gastric GISTs may have more favorable outcomes than Western patients.

In the present study, 5-year RFS rates according to the NIH consensus criteria were 100% in the very low risk, 100% in the low risk group, 88.9% in the intermediate risk group, and 80.1% in the high risk group; those according to the 7th UICC/AJCC TNM staging system were 98.7% in stage I, 93.1% in stage II, 74.1% in stage IIIA, and 60% in stage IIIB. These results are similar to those reported in previous Eastern studies [13, 17], and superior to those reported in Western studies [12, 14, 23, 24].

We evaluated the applicability of the 7th UICC/AJCC TNM staging system as compared to the NIH consensus criteria. The goodness of fit of the 7th UICC/AJCC TNM staging system was higher than that of the NIH consensus criteria, although no significant difference was observed. In a previous study comparing the 7th UICC/AJCC TNM staging system and the NIH consensus criteria, although the authors did not use a statistical method to compare both systems as in our study, they suggested that the 7th UICC/AJCC TNM staging system could reflect the 5-year RFS better than the NIH consensus criteria [13]. Therefore, although no significant difference was observed between these two systems, the 7th UICC/AJCC TNM staging system may be more useful in risk categorization in patients with gastric GISTs than the NIH consensus criteria.

Our study has some limitations. First, the present study was a single-center retrospective study and, as a result, there might have been potential selection biases. Treatment options were selected on a case-by-case basis according to clinical judgment and patient factors. Second, our study had a relatively small number of patients and a short follow-up period. Further large-scale studies with longer follow-up periods are needed to clarify the clinical efficacy of the 7th UICC/AJCC TNM staging system. Third, because we focused only on clinical outcomes in patients who underwent curative resection for gastric GISTs, we did not include patients who had distant metastases at the time of surgery or who underwent preoperative or postoperative chemotherapy. Lastly, we included patients with only gastric GISTs; our results ought to be validated in studies that include GISTs in all sites. The clinical efficacy of the 7th UICC/AJCC TNM staging system and that of the NIH consensus criteria can then be compared according to the tumor’s primary anatomic site.

## Conclusions

In conclusion, the 5-year RFS rate in patients who underwent curative resection for gastric GISTs was excellent. Recurrence occurred in 6.2% of patients with gastric GISTs and was associated with tumor size, mitotic count, and histologic subtype. For risk categorization, the 7th UICC/AJCC TNM staging system may be more useful than the NIH consensus criteria. Further large-scale, multi-center studies with longer follow-up periods are needed to validate our results.

## Abbreviations

AIC: Akaike’s Information Criterion
CI: confidence interval
GISTs: gastrointestinal stromal tumors
HPF: high-power fields
HR: hazard ratio
NIH: National Institutes of Health
RFS: recurrence-free survival
SBC: Schwarz Bayesian Criterion
TNM: tumor-node-metastasis
UICC/AJCC: Union for International Cancer Control/American Joint Committee on Cancer
